# A bibliometric analysis of acupuncture treatment and cognitive impairment

**DOI:** 10.3389/fneur.2025.1495191

**Published:** 2025-06-06

**Authors:** Xiaoqi Yu, Fuchang Lu, Jinlong Chen, Xuanjun Liu, Qingpei Chen

**Affiliations:** ^1^Department of Geriatric Neurology, Guangzhou First People’s Hospital, The Second Affiliated Hospital of South China University of Technology, Guangzhou, China; ^2^The Second Clinical College of Guangzhou University of Chinese Medicine, Guangzhou University of Chinese Medicine, Guangzhou, China; ^3^Medical Big Data Center, Guangdong Provincial People’s Hospital (Guangdong Academy of Medical Sciences), Southern Medical University, Guangzhou, China

**Keywords:** cognition, geriatrics, neurodegenerative diseases, Alzheimer’s disease, Parkinson’s disease, dementia, neurology, CiteSpace

## Abstract

Cognitive impairment, a prevalent neurological disorder characterized by multisystem dysregulation within the nervous system, has prompted substantial scientific inquiry into complementary therapies. This scientometric investigation systematically examines the evolving bilingual (Chinese-English) research paradigm of acupuncture interventions for cognitive impairment through comparative analysis of 510 publications from the China National Knowledge Infrastructure (CNKI) and 633 articles from Web of Science Core Collection, processed via CiteSpace 6.4.R2. Our multidimensional analysis reveals three principal dimensions: (1) Spatiotemporal evolution demonstrating that scholarly contributions in this domain are predominantly clustered within China. Longitudinal bibliometric analysis demonstrates sustained scholarly productivity in this domain, with annual bilingual (Chinese-English) publication outputs consistently exceeding 40 peer-reviewed articles per annum throughout the 2000–2025 observation window, establishing a robust baseline for continuous knowledge advancement; (2) Network analysis atlas of the research institutions and authors reveals that both research output density and institutional affiliations concentrated in Chinese academic hubs and most authors come from China; (3) Divergent thematic trajectories between linguistic cohorts - Chinese studies emphasize vascular mechanisms, oxidative stress modulation, and pharmacological synergies, whereas English literature prioritizes gut-brain axis interactions, postoperative cognitive recovery, and neuroinflammatory pathways. These findings provide evidence-based insights into acupuncture’s therapeutic mechanisms in cognitive impairment while establishing a conceptual framework to guide future translational studies and clinical protocol optimization in integrative neurology.

## 1 Introduction

Cognitive impairment is a common neurological disorder (1). One or multiple functions including memory, language, visuospatial, execution, calculation and judgement have been found impaired in cognitive impairment patients (2). It is predicted that the incidence of cognitive impairment will continue to increase to 159.2 million cases worldwide by 2025 according to existing research, which places a heavy burden on individuals, families, and society (3).

Different treatments are available for cognitive impairment due to different etiologies. NMDAR antagonist memantine has some effect on cognitive impairment caused by schizophrenia while acetylcholinesterase inhibitors such as AChEIs, Donepezil, Rivastigmine and Galantamine are mainly aimed at dementia (4, 5). The efficacy of medications for major depressive disorder, for example, Citalopram, Duloxetine, Desvenlafaxine, Venlafaxine, and Sertraline is still unclear because the sample size of the studies is insufficient and the samples are mainly consisted with elderly, missing samples for other ages (6). In addition to these conventional medications, a number of new antidepressants such as Vortioxetine and Ketamine have been identified that can be used to treat cognitive impairment recently. But Vortioxetine only has effect on enhancing cognition while Ketamine still requires more longitudinal replication and active-comparator trials (6). So far, there has been still no specific drugs for cognitive impairment.

However, in addition to drug treatment, a large number of studies find that acupuncture is effective in treating cognitive impairment (7–12). Acupuncture activates neuroprotective functions through blocking the aggregation of toxic proteins in neurological diseases, inhibiting neurotoxicity and modulating glucose metabolism (7). A randomized, controlled, parallel-group, exploratory study comparing acupuncture to donepezil hydrochloride finds that acupuncture is associated with better efficacy and no adverse events (8). Evidence from a meta-analysis shows that acupuncture is better than medication in improving mental state (13). Moreover, acupuncture is more effective when combined with other medications. Acupuncture combines with donepezil is more effective than donepezil alone at improving the mini-mental state examination (MMSE) scale score (12). Above all, acupuncture alone or in combination with other medications has a significant effect on cognitive impairment.

Bibliometrics refers to the interdisciplinary science that uses mathematical and statistical methods to quantitatively analyze all knowledge carriers (14). CiteSpace is currently the most widely used visual analysis tool for bibliometric studies coded by Prof. Chaomei Chen. It can be used to analyze research trends and recent developments in a certain research field and present them in a visual form (15).

Current bibliometric syntheses addressing acupuncture interventions for cognitive impairment remain constrained by temporal parameters terminating in 2022, thereby excluding critical advancements documented during the recent triennium (2023–2025) (16). This temporal discontinuity creates substantive gaps in characterizing evolving research trajectories, emergent transnational collaboration patterns, and paradigm shifts toward precision neuromodulation approaches. Our systematic review bridges this epistemological divide through rigorous analysis of 1,143 peer-reviewed publications (Web of Science Core Collection/CNKI) spanning 2000–2025, processed via CiteSpace 6.4.R2 with strict adherence to PRISMA-ScR guidelines. By incorporating the latest research data and methodological innovations in neuroimaging-coupled acupuncture research, this temporal extension enables: (1) quantification of shifting geographic productivity cores beyond established Chinese academic hubs; (2) evidence-based stratification of knowledge domains requiring urgent translational investigation. These temporal-expansion-driven insights establish a dynamic framework for benchmarking progress in this rapidly evolving therapeutic domain while addressing the critical need for updated scientometric baselines to inform global research prioritization.

## 2 Materials and methods

### 2.1 Data collection

China National Knowledge Infrastructure (CNKI) database was used for Chinese literature data retrieval. The search formula is: SU = (‘针’ + ‘针灸’ + ‘灸’ + ‘电针’ + ‘毫针’ + ‘火针’ + ‘腕踝针’ + ‘眼针’ + ‘揿针’ + ‘蜂针’ + ‘舌针’ + ‘腹针’ + ‘耳针’ + ‘头针’ + ‘体针’ + ‘针法’ + ‘艾灸’) AND SU = (‘认知障碍’ + ‘轻度认知障碍’ + ‘轻度认知损伤’ + ‘轻度认知损害’ + ‘MCI’ + ‘SCD’). Totally 1,210 publications were collected. Conference abstract (*n* = 49), dissertations (*n* = 415), books (*n* = 1) and achievement (*n* = 14) were excluded. Following a two-stage screening protocol involving title/abstract triage and full-text evaluation against predefined inclusion criteria, 221 non-conforming articles were systematically excluded, resulting in a final analytical cohort of 510 rigorously vetted publications spanning the study period.

Science Citation Index Expand (SCI-E) database of Web of Science Core Collection was used for English literature data retrieval. The search formula is: TS = (acupuncture OR acupuncture therapy OR electroacupuncture OR moxibustion therapy) AND TS = (mild cognitive impairment OR cognitive impairment OR cognitive dysfunctions OR cognitive disorder OR cognitive decline OR mental deterioration OR dementia). Review article (*n* = 240), meeting abstract (*n* = 11), editorial material (*n* = 8), early access (*n* = 7), retracted publication (*n* = 7), correction (*n* = 5), book chapters (*n* = 3), proceeding paper (*n* = 3), letter (*n* = 2) and retraction (*n* = 2) were excluded. A dual-phase filtration process comprising algorithmic keyword screening and manual full-text appraisal eliminated 76 irrelevant records, yielding a curated corpus of 633 high-evidence publications that met stringent eligibility thresholds, with inter-rater reliability scoring confirming robust selection validity (*κ* = 0.86) across the defined temporal spectrum (2000–2025).

**Figure 1:**
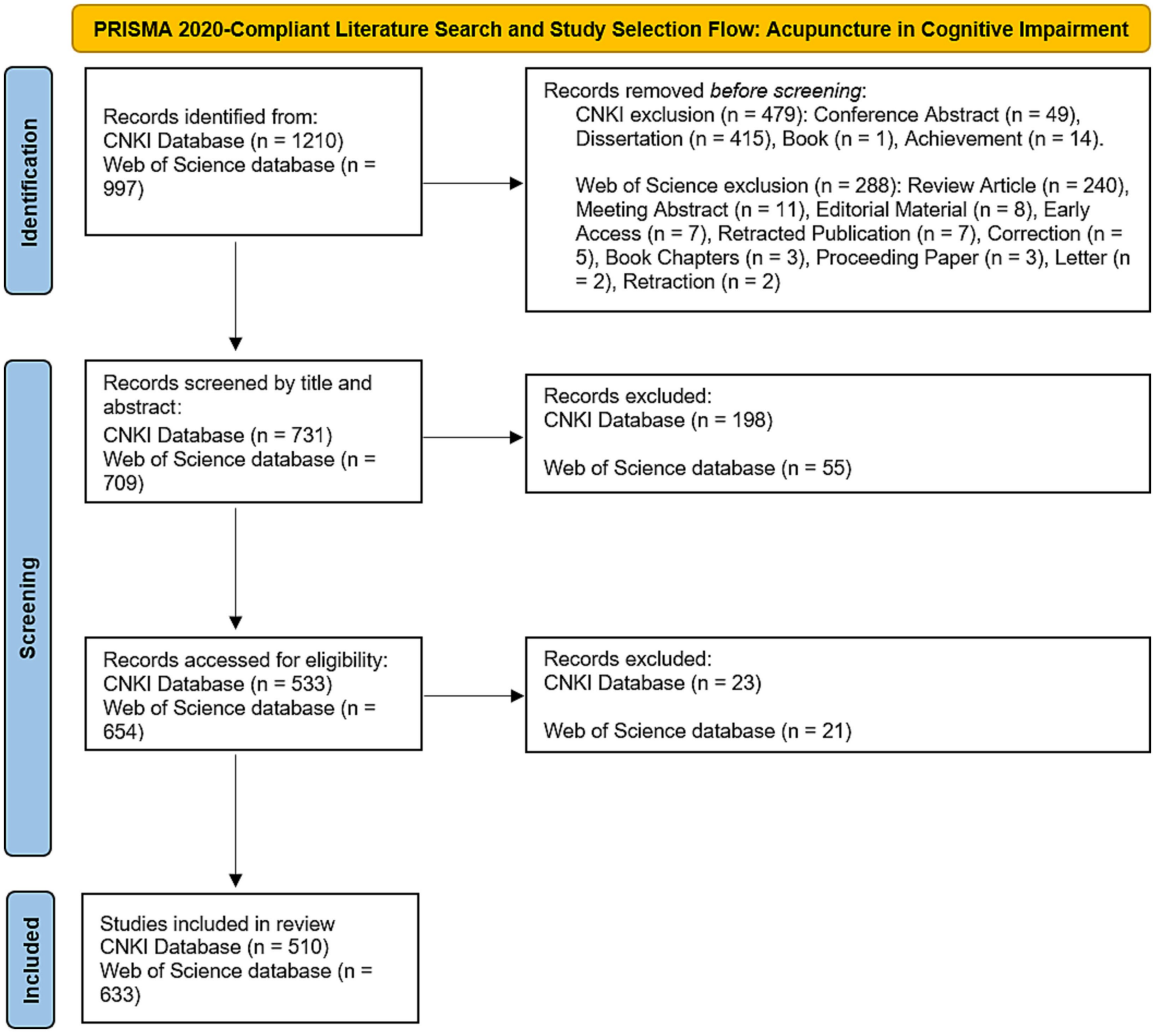
Flowchart illustrating the literature search and screening process for studies on acupuncture in cognitive impairment. Initial searches identified 1,210 records from the China National Knowledge Infrastructure (CNKI) database and 997 records from Web of Science. Exclusion criteria were applied to remove non-eligible publication types: 479 records from CNKI (conference abstracts: 49; dissertations: 415; books: 1; achievements: 14) and 331 records from Web of Science (review articles: 288; meeting abstracts: 11; editorials: 8; early access articles: 7; retracted publications: 7; corrections: 5; book chapters: 3; proceeding papers: 3; letters: 2; retractions: 2). After full-text assessment, 510 studies from CNKI and 585 studies from Web of Science were included for bibliometric analysis, adhering to predefined eligibility criteria for methodological rigor and thematic relevance.

Both the Chinese and English literature were collected spanning their inception to March 2025. Figure 1 shows the flowchart for search strategy and selection process in this study.

### 2.2 Data processing

CiteSpace is a widely used java-based bibliometric tool developed by Prof. Chaomei Chen of Drexel University in the United States. It is a visual bibliometric analytics tools for network analysis, cluster analysis and spatiotemporal analysis of countries, institutions, authors, journals and keywords. Its core functionality rests on three analytical pillars: co-occurrence analysis, citation network mapping, and burst detection, supported by graph theory and information visualization principles. In this article, 510 Chinese and 633 English selected publications were analyzed by CiteSpace 6.4.R2. software to display the research progress and frontier about acupuncture and cognitive impairment. Narrative reviews were excluded in this review, but meta-analyses that followed systematic review methods (e.g., PRISMA) were included as they provided quantitative data synthesis and effect size calculations, which were consistent with the study objectives. Standardized bibliographic extraction protocols were implemented, with Chinese-language records from the China National Knowledge Infrastructure (CNKI) exported in RefWorks format and sequentially renamed as download_1.txt, download_2.txt, etc., while English publications retrieved from Web of Science Core Collection were exported as plain text files containing full metadata records and cited references, following identical alphanumeric nomenclature for cross-platform consistency. This dual-channel archiving strategy ensured format-specific data integrity while maintaining parity in downstream computational processing pipelines.

### 2.3 Bibliometric analysis

Network analyses of geopolitical, institutional, and authorship collaboration patterns were conducted using CiteSpace 6.4.R2 with the following parameterization: temporal scope spanning database inception through March 2025 (1-year segmentation intervals), node extraction from title/abstract fields supplemented by author-generated keywords (DE) and indexer-assigned keywords (ID). The g-index (*k* = 25) was applied to optimize network resolution, with pathfinder pruning algorithms implemented to eliminate redundant co-occurrence links while preserving structural integrity of sliced networks. For semantic mapping analyses – including keyword clustering, citation burst detection, and temporal trajectory visualization – node typology was restricted to keyword entities. This dual-layer analytical framework enables simultaneous examination of macro-level collaborative architectures and micro-level conceptual evolution through computationally validated scientometric workflows.

### 2.4 Definition and scope of terminology

Cognitive impairment refers to a spectrum of conditions characterized by measurable declines in cognitive functions—such as memory, attention, language, or executive function—that exceed age-related expectations but do not yet severely disrupt daily activities. Dementia represents the most advanced subset of cognitive impairment, primarily affecting older adults. Neurodegeneration denotes the progressive loss of neuronal structure or function, underlying diseases such as Alzheimer’s Disease (AD), Parkinson’s disease, and Huntington’s disease. While not all cognitive impairment stems from neurodegeneration, this review focuses on acupuncture’s role in neurodegenerative cognitive disorders, particularly AD and related dementias. Cognitive impairment serves as the umbrella term, with dementia representing its severe manifestation. Neurodegeneration constitutes a primary etiological pathway for dementia.

## 3 Results

### 3.1 Bibliometric analysis of Chinese literature

#### 3.1.1 Temporal dynamics of acupuncture research on cognitive impairment of Chinese literature in CNKI database

Longitudinal publication trends serve as critical scientometric indicators for assessing disciplinary maturation and forecasting emergent research trajectories. Quantitative temporal analysis of scholarly output, when coupled with polynomial regression modeling, enables systematic evaluation of developmental phases and inflection points within evolving academic domains.

**Figure 2:**
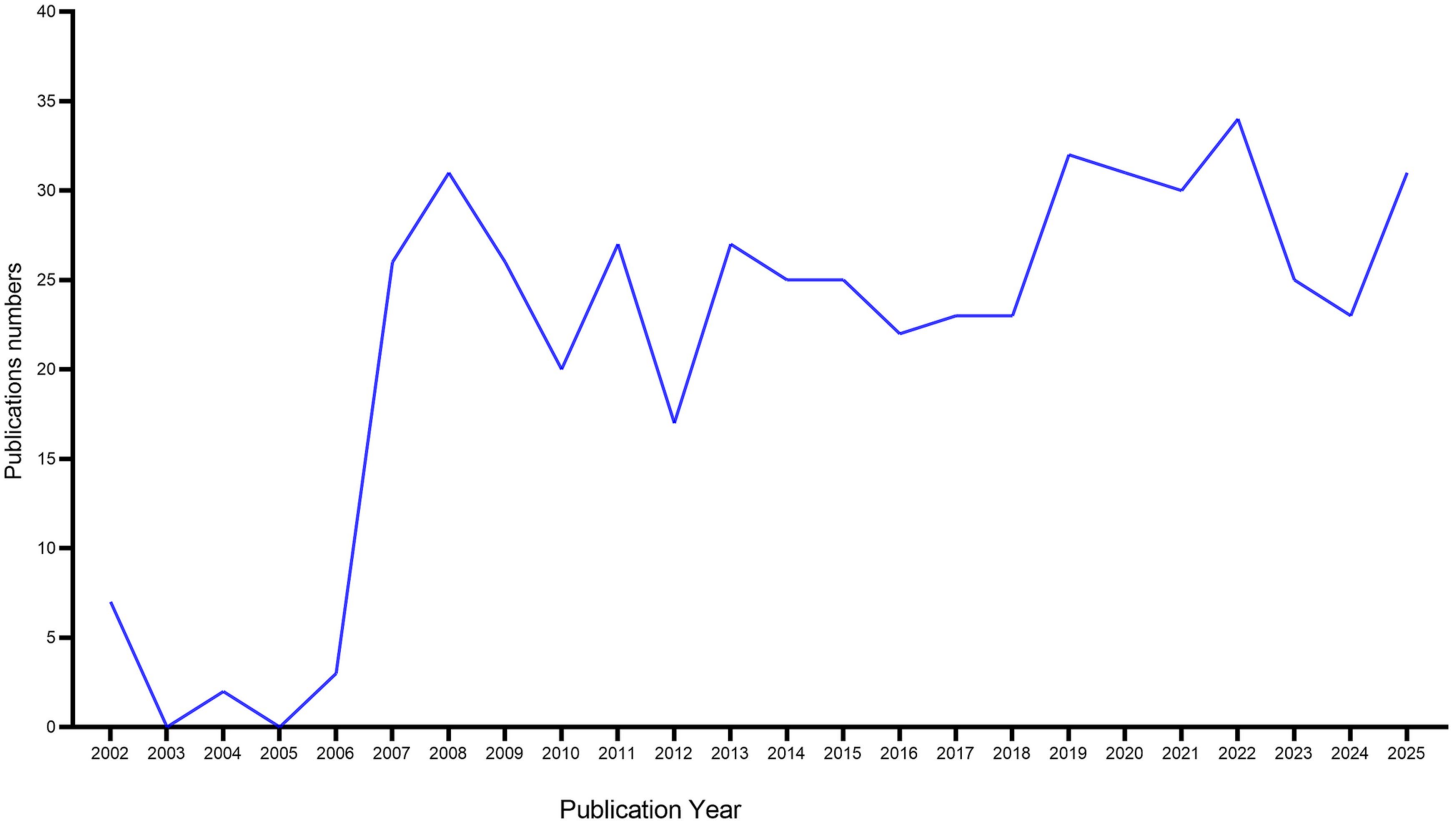
Trends in the growth of publications of Chinese literature in CNKI database. Number of publications over 23 years were displayed.

As illustrated in Figure 2, our analysis of 510 CNKI-indexed publications reveals a triphasic growth pattern in acupuncture research targeting cognitive impairment: Incubation Phase (2002–2006): Limited academic engagement, reflecting nascent conceptual exploration. Exponential Growth Phase (2006–2008): Demonstrated a surge in annual output. Consolidation Phase (2009–2025): Stabilized productivity with enhanced methodological sophistication, indicating paradigm transition towards evidence-based clinical translation.

#### 3.1.2 Network analysis of research institutions and authors in CNKI database

**Figure 3:**
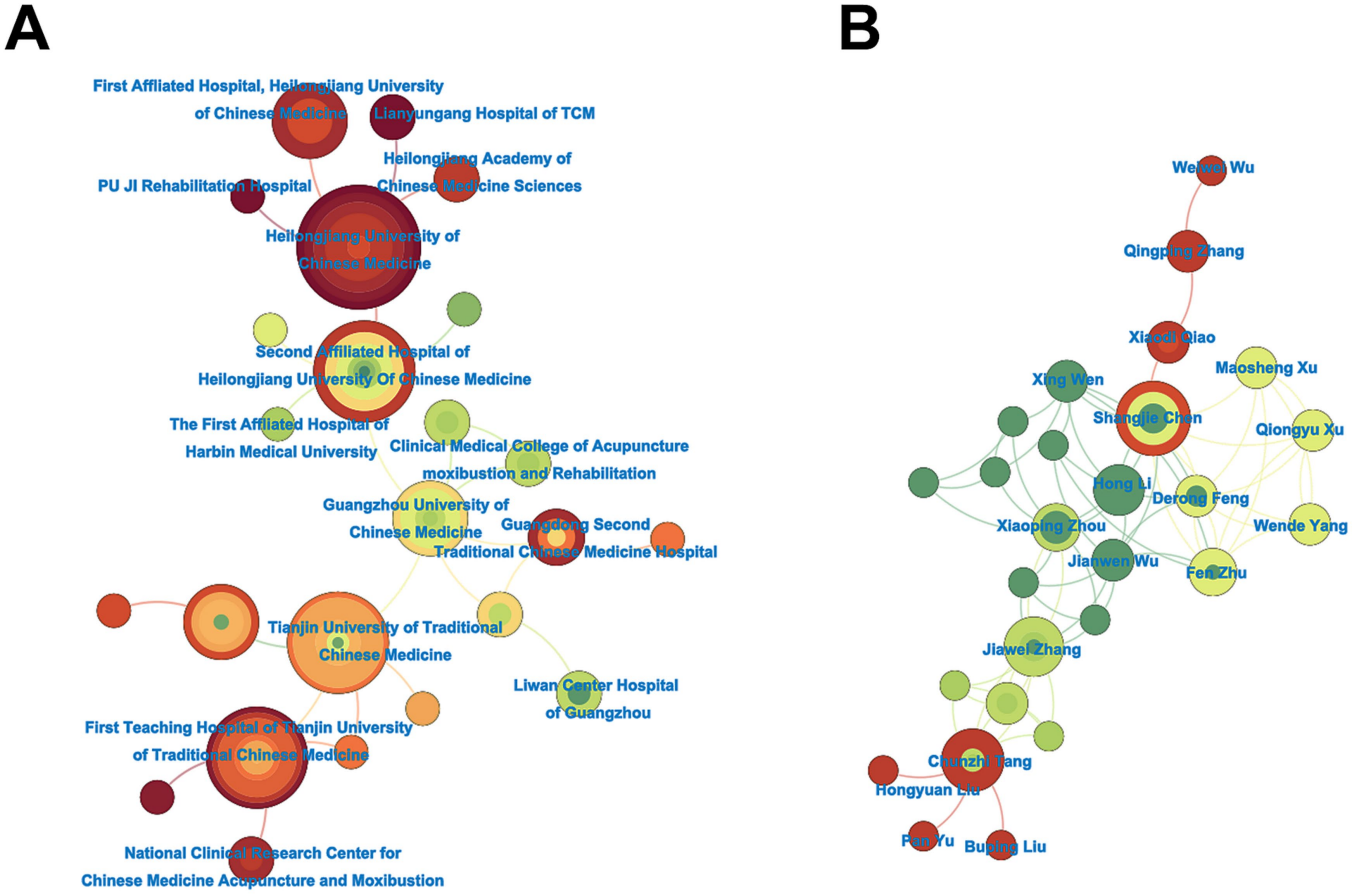
Network analysis atlas of the research institutions and authors in CNKI database. Network analysis of research institutions **(A)** and authors **(B)** in acupuncture-related cognitive impairment studies from the CNKI database, generated using CiteSpace 6.4.R2. Node size reflects publication volume, with larger nodes indicating higher productivity. Line thickness denotes collaboration frequency, highlighting robust inter-institutional partnerships. Color gradients represent temporal activity, with warmer hues (red/orange) indicating recent contributions.

Figure 3A delineates the structural topology of institutional collaborations through a force-directed network visualization generated by CiteSpace 6.4.R2. The network exhibits a scale-free distribution with Heilongjiang University of Chinese Medicine serving as the primary hub. This nodal prominence reflects its dual role in coordinating: sustained partnerships with PU JI Rehabilitation Hospital and high-density connections with Lianyungang Hospital of TCM and Heilongjiang Academy of Chinese Medicine Sciences. Quantitative dominance is concentrated among Northeast Chinese institutions: Heilongjiang University of Chinese Medicine (16 papers), followed by Tianjin University of Traditional Chinese Medicine (9 papers) and First Teaching Hospital of Tianjin University of Traditional Chinese Medicine (9 papers).

Figure 3B delineates the co-authorship network topology through modularity-driven clustering analysis, revealing three dominant collaborative paradigms. First, Prof. Shangjie Chen emerges as the pivotal knowledge broker, coordinating a multidisciplinary consortium spanning: sustained collaboration with Xiaodi Qiao, Xing Wen, Li Hong and Derong Feng. Dr. Chunzhi Tang also anchors a cluster with Hongyuan Liu, Pan Yu and Buping Liu.

#### 3.1.3 Keyword analysis in CNKI database

**Figure 4:**
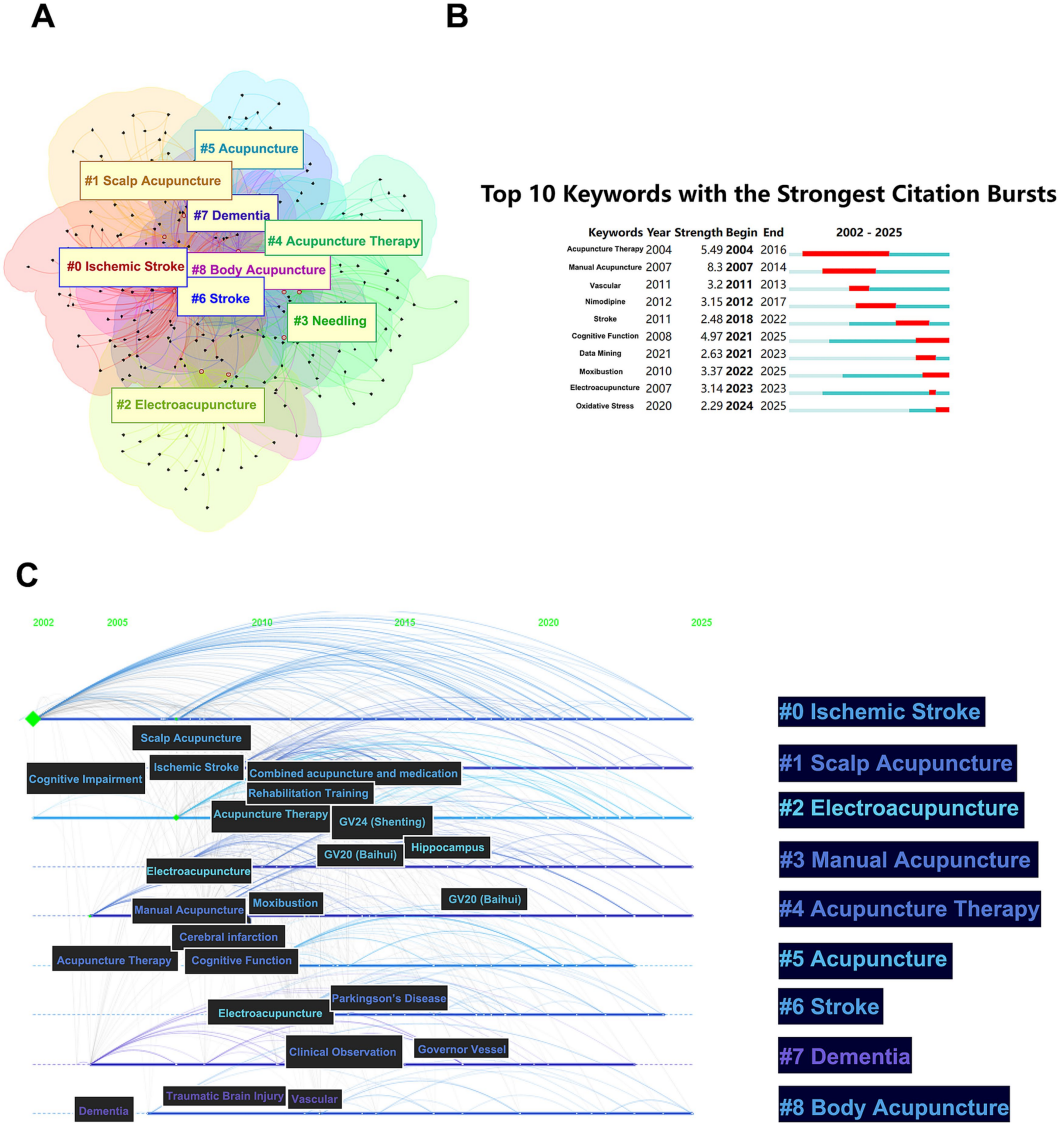
CNKI Keyword cluster map, present map and time line chart. **(A)** Keyword co-occurrence cluster map of acupuncture research in cognitive impairment from the CNKI database, generated using CiteSpace 6.4.R2 with log-likelihood ratio (LLR) clustering. **(B)** Top 10 keywords with the strongest citation bursts in acupuncture-related cognitive impairment research, generated via CiteSpace 6.4.R2 using Kleinberg’s burst detection algorithm (*γ* = 0.5). Bar length indicates burst duration, while height reflects burst strength. Red segments highlight active citation surge periods. **(C)** Temporal evolution of keyword clusters in acupuncture-related cognitive impairment research from the CNKI database (2002–2025), visualized using CiteSpace 6.4.R2 with a temporal slicing algorithm (5-year intervals).

The keyword co-occurrence network (Figure 4A) reveals a modular knowledge structure comprising nine thematic clusters that map the epistemological evolution of this field: core therapeutic concepts (#4 Acupuncture Therapy, #5 Acupuncture), pathology-specific interventions (#0 Ischemic Stroke, #6 Stroke, #7 Dementia) and technical methodologies (#1 Scalp Acupuncture, #2 Electroacupuncture, #3 Needling, #8 Body Acupuncture).

Burst detection analysis (Figure 4B) quantifies three distinct innovation waves: Wave 1 (2004–2007): dominated by “acupuncture therapy,” reflecting foundational clinical validation efforts. Wave 2 (2008–2012): Emergence of “cognitive function” correlating with acupuncture studies on cognitive function. Wave 3 (2020–2021): “oxidative stress” and “Data mining” convergence with data analysis and oxidative stress mechanism research.

Temporal trajectory mapping (Figure 4C) delineates a paradigm shift across three epochs: Symptom-Centric Phase (2002–2010): keywords focused on general efficacy evaluation (e.g., “cognitive impairment,” “acupuncture therapy”). Targeted Mechanistic Phase (2011–2015): Neural circuit mapping terms (“hippocampus,” “Parkingson’s Disease”). Precision Integration Phase (2016–2025): Emergence of “GV24 (Shenting)” and “GV20 (Baihui).

### 3.2 Bibliometric analysis of English literature

#### 3.2.1 Temporal dynamics of acupuncture research on cognitive impairment of English literature in web of science database

**Figure 5:**
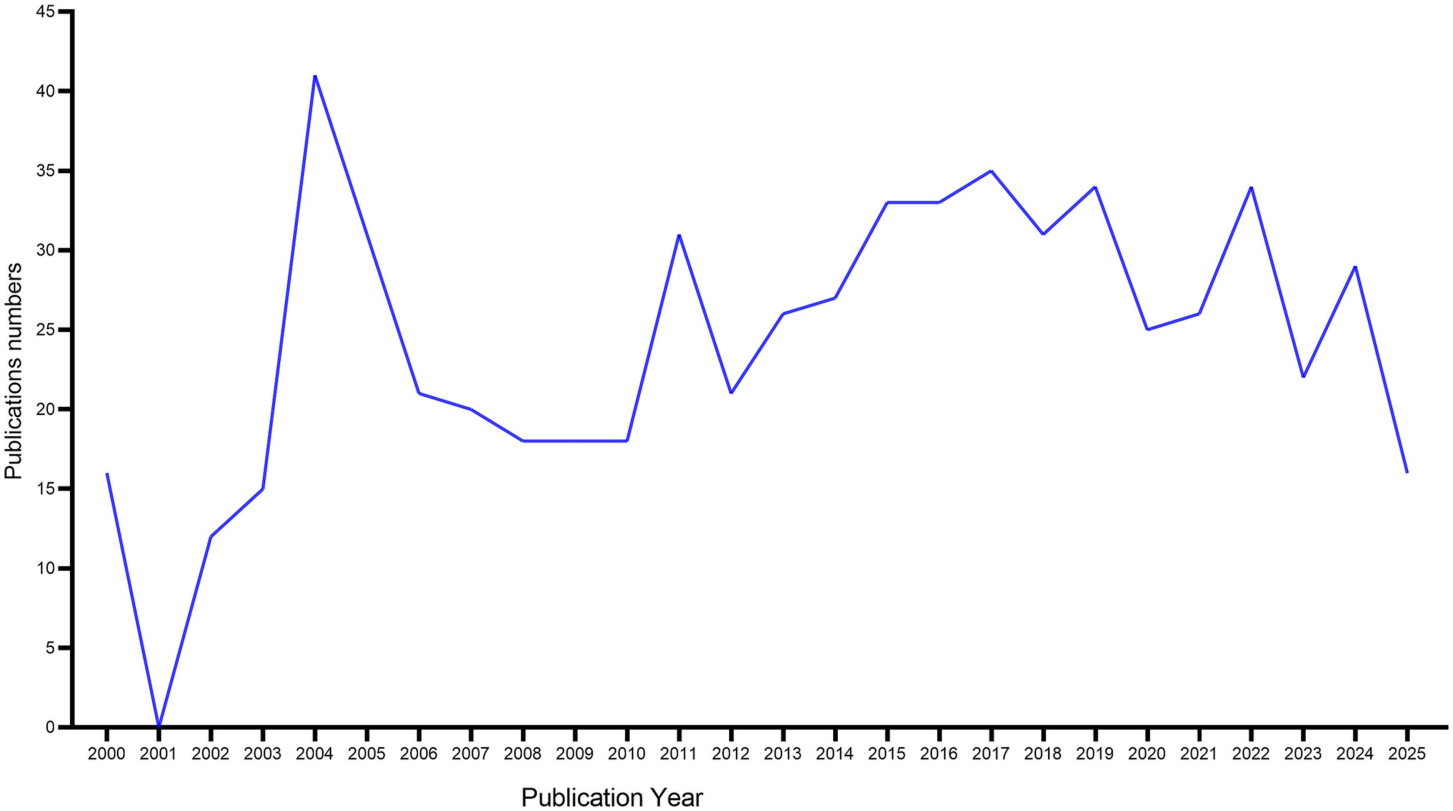
Trends in the growth of publications of English literature in Web of Science database. Number of publications over 25 years were displayed.

Quantitative analysis of 633 Web of Science Core Collection-indexed publications reveals three distinct developmental epochs (Figure 5), each demarcated by critical inflection points in research output and methodological innovation: Incubation phase (2000–2003): Limited academic engagement, less than 20 publications per year. Exponential growth phase (2004–2017): 2-fold output surge. Consolidation phase (2018–2025): sustained scholarly output with a stabilized annual productivity threshold exceeding 20 peer-reviewed articles, indicating robust research continuity despite methodological paradigm shifts toward multi-omics integration and AI-driven clinical trial designs.

#### 3.2.2 Distribution of countries/regions

**Figure 6:**
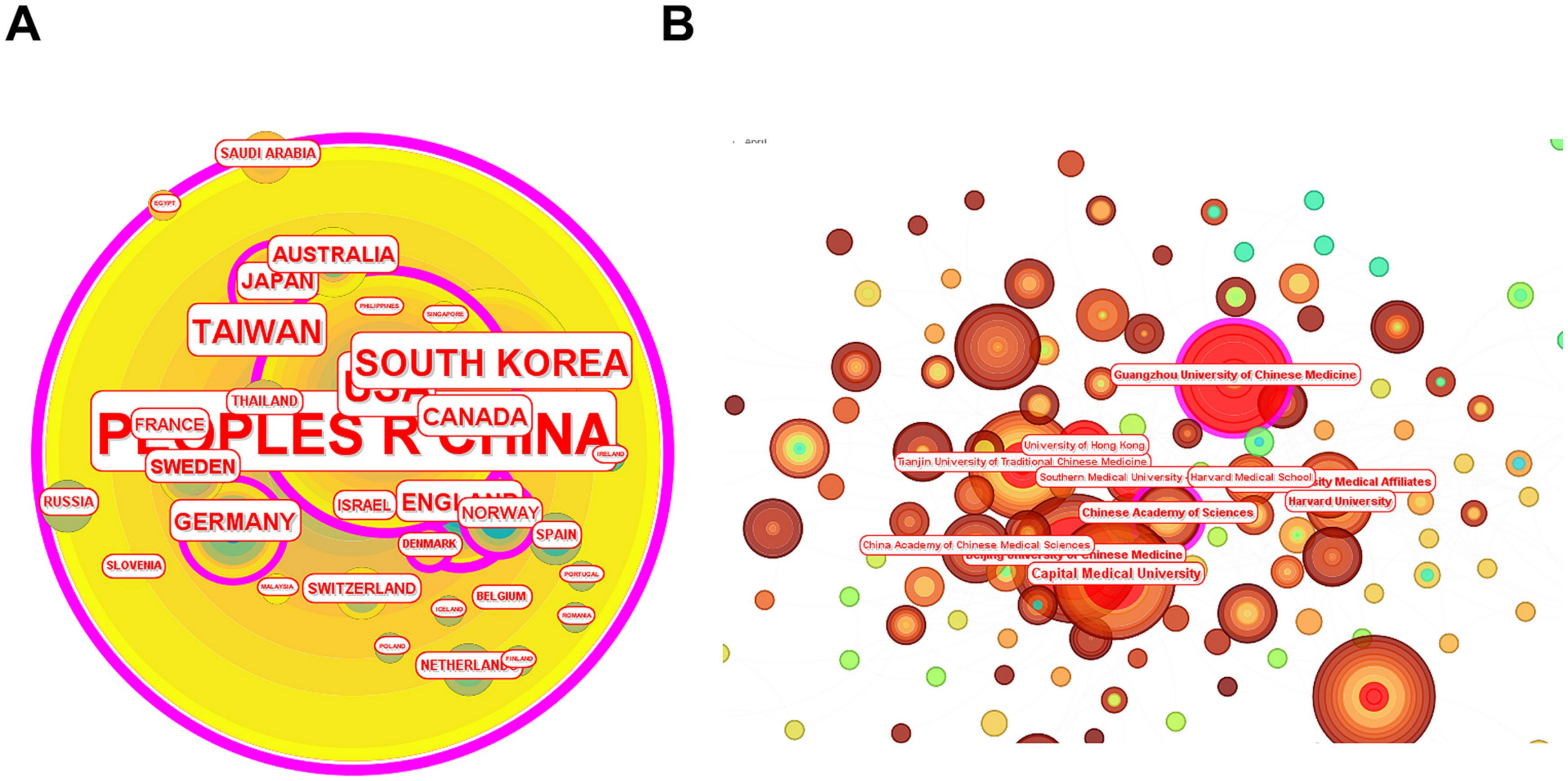
Network analysis atlas of the country/region and research institutions in Web of Science database. This network visualization map, constructed using CiteSpace 6.4.R2, illustrates collaborative patterns among countries **(A)** and research institutions **(B)** based on publications retrieved from the Web of Science database. Nodes represent countries or institutions, with size proportional to their publication output. Colors distinguish clusters of closely linked entities.

The geopolitical collaboration network (Figure 6A), constructed via CiteSpace 6.4.R2 using a modified Fruchterman-Reingold algorithm, quantitatively delineates international research dynamics through these primary topological metrics: Node diameter correlates with academic output volume, revealing China’s dominance. Betweenness centrality (BC ≥ 0.15) identifies China (BC = 0.61), USA (BC = 0.24) and England (BC = 0.24) as global knowledge intermediaries. CIELAB color space encoding tracks collaboration chronology: Emerald represents early-stage contributions, while vermilion reflects recent activity burst. Key findings reveal two dominant clusters: Asian collaborations: China maintains strong partnerships with South Korea, Japan, and Thailand, supported by regional initiatives. European collaborations: Germany and France form a core partnership while scandinavian countries (Sweden, Norway, Denmark) form another partnership.

#### 3.2.3 Distribution of research institution

The institutional collaboration network depicted in Figure 6B was generated through CiteSpace 6.4.R2 (time slicing: 2000–2025, interval = 1 year). Key analytical dimensions include: Node size: Proportional to the cumulative publication output of each institution. Chronological activity: Node color gradient reflects temporal collaboration intensity, with red/orange hues denoting institutions exhibiting sustained collaborative activity. Prominent contributors include Beijing University of Chinese Medicine (57 papers), Fujian University of Traditional Chinese Medicine (54 papers), Capital Medical University (52 papers), Guangzhou University of Chinese Medicine (49 papers) and Tianjin University of Traditional Chinese Medicine (33 papers) (Table 1). These institutions demonstrate both high publication volumes and recent surges in collaborative engagement. Notably, most of nodes originate from Chinese institutions, highlighting China’s pivotal role as the primary research hub in this domain.

**Table 1:** Top 10 institutions in acupuncture and cognitive impairment.

Rank	Record count	Institution
1	57	Beijing University of Chinese Medicine
2	54	Fujian University of Traditional Chinese Medicine
3	52	Capital Medical University
4	49	Guangzhou University of Chinese Medicine
5	33	Tianjin University of Traditional Chinese Medicine
6	29	Korea Institute of Oriental Medicine (KIOM)
7	26	Shanghai University of Traditional Chinese Medicine
8	20	China Medical University Taiwan
9	18	Chinese Academy of Sciences
10	17	China Academy of Chinese Medical Sciences

#### 3.2.4 Author collaboration network analysis

**Figure 7:**
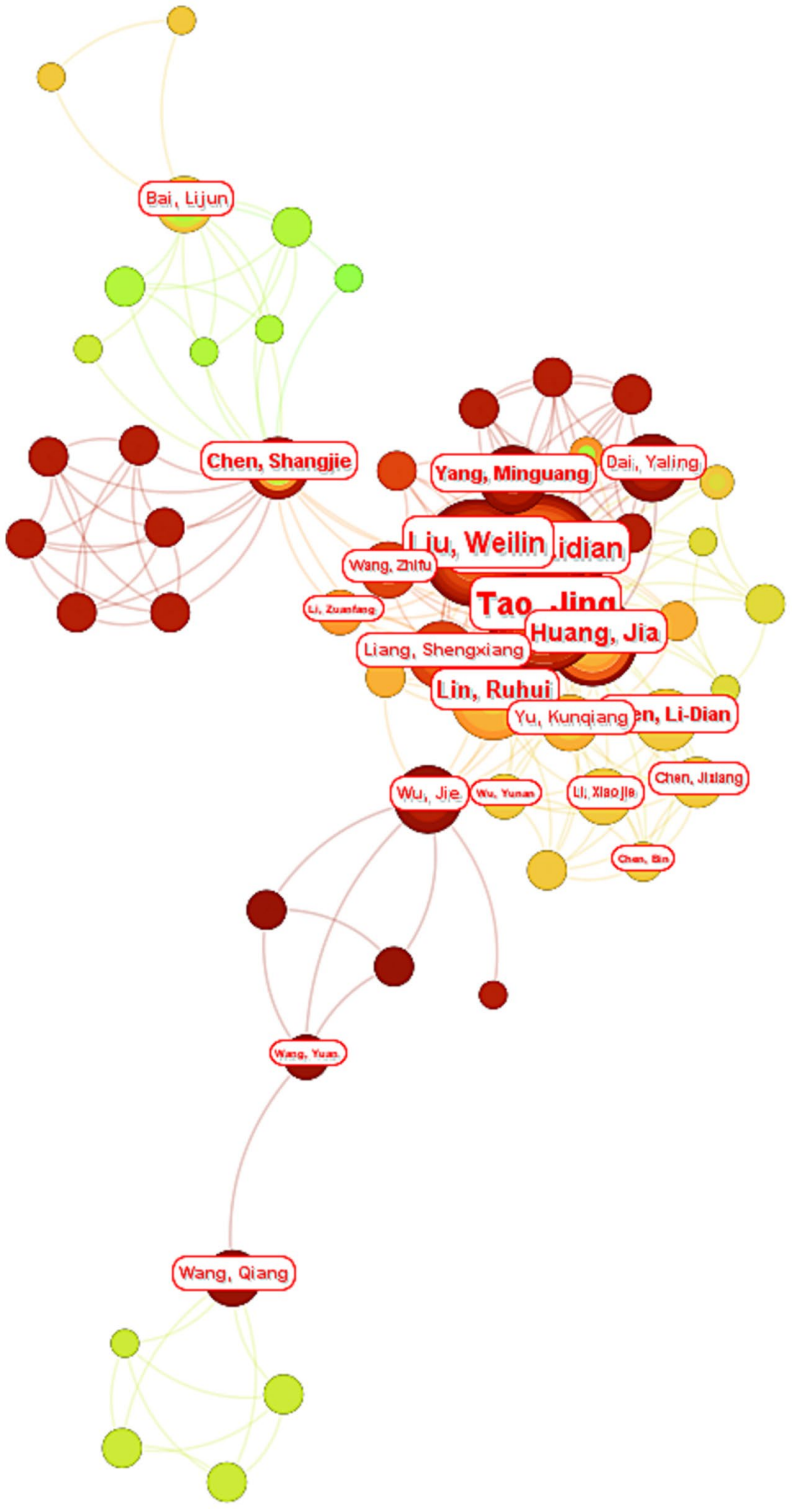
Network analysis atlas of the authors in Web of Science database. This co-authorship network map, generated using CiteSpace 6.4.R2, visualizes collaborative relationships among authors based on publications retrieved from the Web of Science database. Nodes represent individual authors, with their size corresponding to publication productivity. Connecting lines indicate co-authorship frequency, where thicker lines reflect stronger collaborative ties. Distinct colors demarcate clusters of authors with shared collaborative patterns.

The co-authorship network (Figure 7), constructed using CiteSpace 6.4.R2 with a hybrid clustering algorithm [log-likelihood ratio (LLR) optimization], delineates collaborative patterns through these analytical axes: Node size reflects proportional to publication output and color gradient represents temporal engagement [RGB: green (2000) to red (2025)]. A tightly interconnected hub dominates the network, comprising six pivotal authors: Tao Jing, Chen Lidian, Huang Jia, Lin Ruhui, Yang Mingguang and Liu Weilin.

#### 3.2.5 Interpretation of journal dual-map overlay

**Figure 8:**
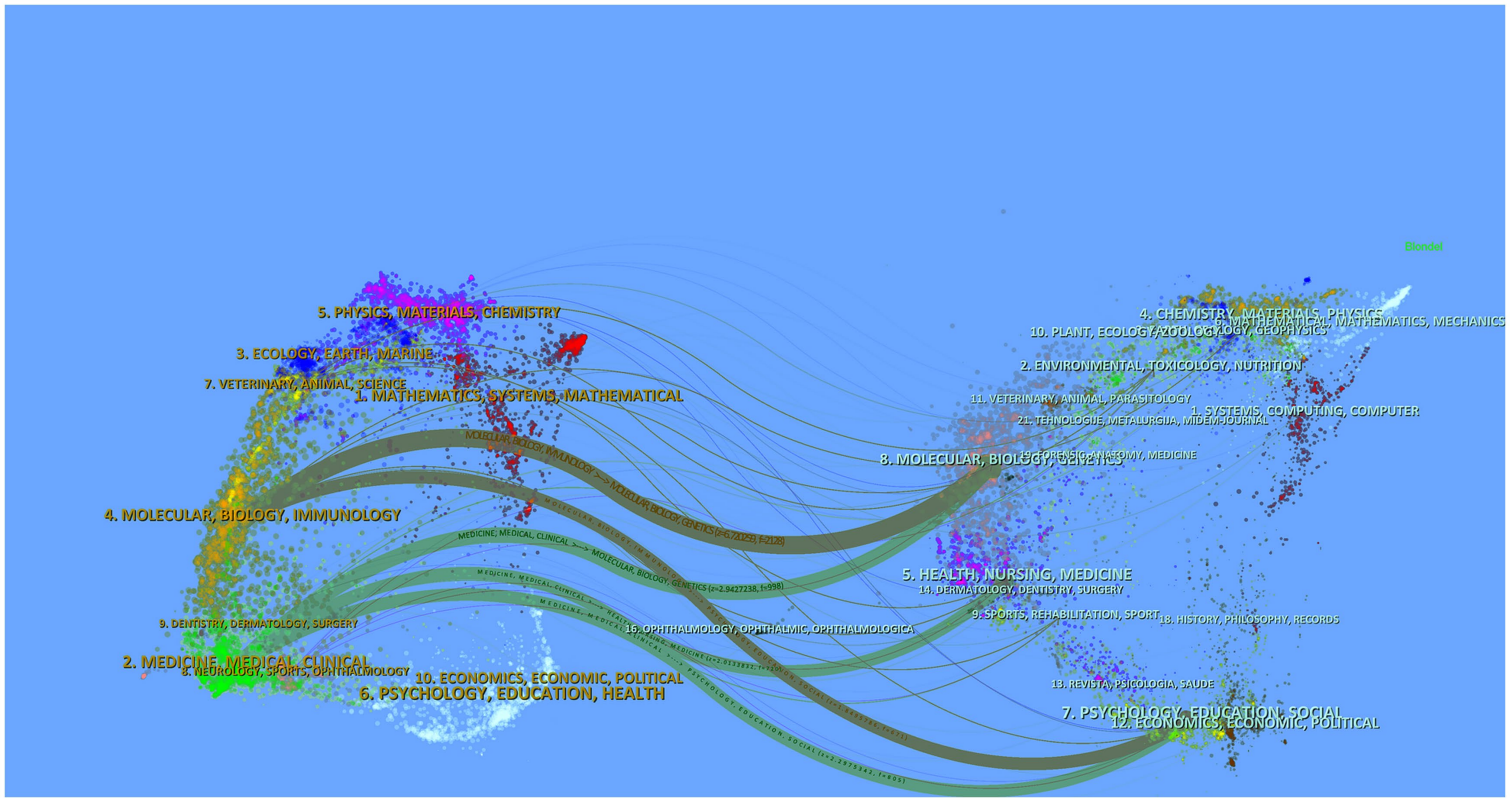
Dual map overlay of journals in Web of Science database. This dual map overlay generated using CiteSpace 6.4.R2 visualizes interdisciplinary citation trajectories between journals based on publications retrieved from the Web of Science database. The left side represents citing journals (sources of publications), while the right side depicts cited journals (targets of references). Colored curves denote citation linkages, with thickness proportional to citation frequency. Journals are clustered by discipline, and their positions reflect thematic relevance. Key citation pathways highlight interdisciplinary interactions.

The dual-map overlay of scientific journals (Figure 8), generated using CiteSpace 6.4.R2, systematically maps interdisciplinary knowledge transfer between citing (left) and cited (right) journal clusters. Dominant clusters include Psychology, Molecular Biology, and Medicine/Physics, where larger ellipses indicate high disciplinary productivity. Core knowledge bases are anchored in Genetics and Health Sciences, with citation trajectories revealing two principal cross-domain patterns: Gray trajectories: citations flow from Molecular Biology/Immunology journals to Psychology/Education/Social Sciences, reflecting translational applications of basic research. Green trajectories: linkages connect Medical/Clinical journals with Molecular Biology/Genetics and Health Nursing, demonstrating clinical-basic science integration.

#### 3.2.6 Research hotspots and frontier analysis

**Figure 9:**
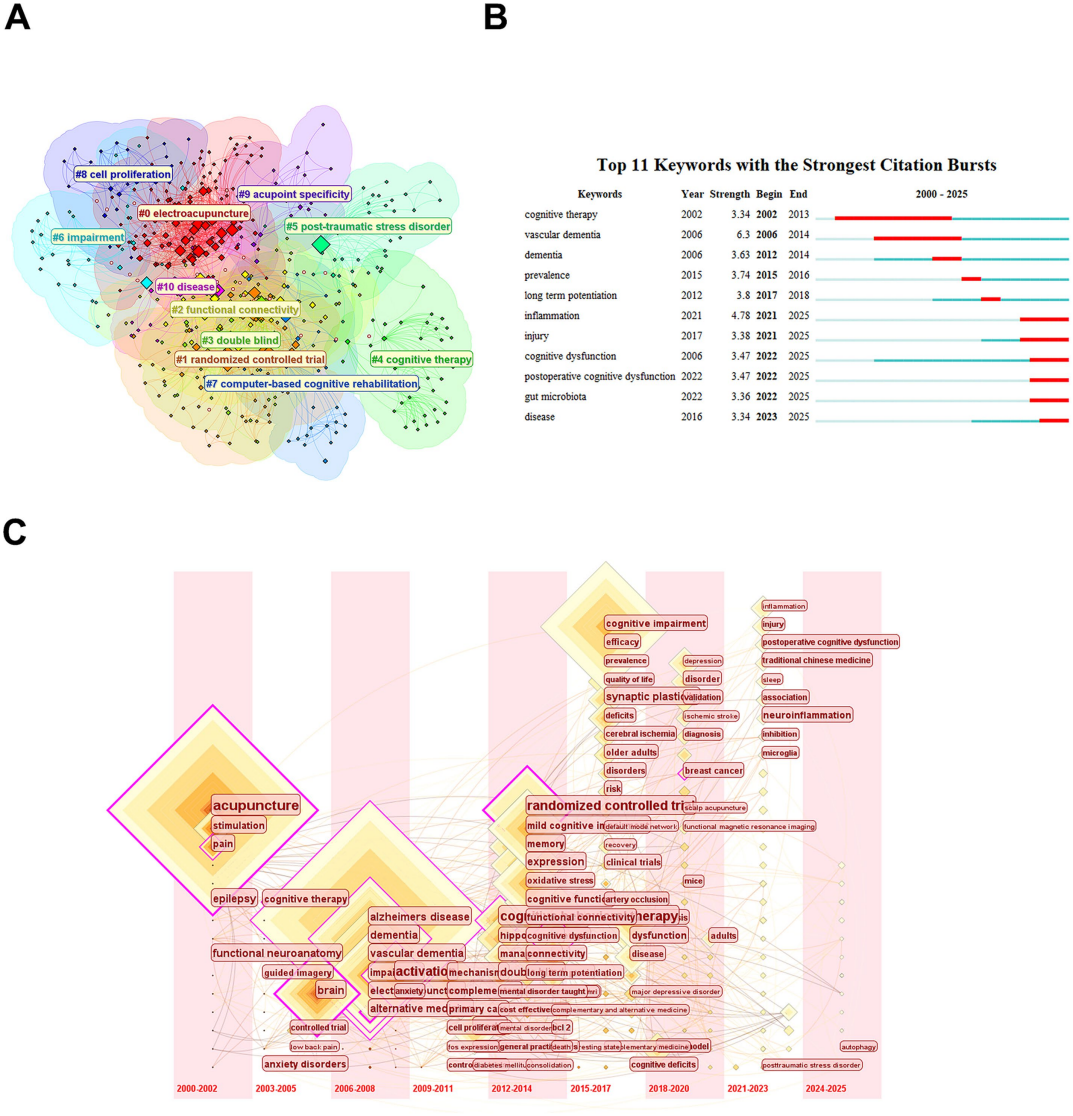
Web of Science keyword cluster map, present map and time line chart. **(A)** Keyword co-occurrence cluster map of acupuncture research in cognitive impairment from the Web of Science database, generated using CiteSpace 6.4.R2 with log-likelihood ratio (LLR) clustering. **(B)** Top 10 keywords with the strongest citation bursts in acupuncture-related cognitive impairment research in Web of Science database, generated via CiteSpace 6.4.R2 using Kleinberg’s burst detection algorithm (γ = 0.5). Bar length indicates burst duration, while height reflects burst strength. Red segments highlight active citation surge periods. **(C)** Temporal evolution of keyword clusters in acupuncture-related cognitive impairment research from the Web of Science database (2000–2025), visualized using CiteSpace 6.4.R2 with a temporal slicing algorithm (2-year intervals).

The keyword co-occurrence cluster map (Figure 9A), constructed using CiteSpace 6.4.R2 with a log-likelihood ratio (LLR) algorithm, reveals the intellectual structure of the field. Node size represents keyword frequency. Cluster labels which highlighting research themes were automatically extracted by LLR. This network identifies 11 thematic clusters ranked by research intensity: #0 electroacupuncture, #1 randomized controlled trial and #2 functional connectivity, #3 double blind, #4 cognitive therapy, #5 post-traumatic stress disorder, #6 impairment, #7 computer-based cognitive rehabilitation, #8 cell proliferation, #9 acupoint specificity, #10 disease. The dominant clusters include #0 electroacupuncture, #1 randomized controlled trial and #2 functional connectivity. The emergent domains contain #7 computer-based cognitive rehabilitation and #9 acupoint specificity.

The citation burst diagram (Figure 9B), generated via CiteSpace 6.4.R2 using Kleinberg’s burst detection algorithm, identifies temporal hotspots and emerging trends in acupuncture-cognitive impairment research. The temporal evolution reveals three evolutionary phases: the early phase (2002–2006) focused on clinical nosology, with “dementia” and “cognitive dysfunction” dominating discourse. During the transition phase (2012–2017), emphasis shifted to epidemiological impact, marked by bursts in “prevalence” (strength = 3.74) and “long term potentiation” (strength = 3.8), coinciding with global burden of disease initiatives. The modern phase (2021–2025) exhibits mechanistic predominance, with “neuroinflammation” (strength = 4.78) and “gut microbiota” (strength = 3.36) reflecting a paradigm shift toward molecular pathways.

The keywords time zone diagram (Figure 9C), constructed using CiteSpace 6.4.R2 with a temporal slicing algorithm (2000–2025, 1-year intervals), maps the evolutionary trajectory of research themes in acupuncture-cognitive impairment studies. This visualization integrates chronological and thematic dimensions through the following analytical elements: Horizontal axis represents time progression segmented into developmental stages. Vertical axis displays keyword clusters grouped by thematic similarity. Node size reflects proportional to keyword frequency. Red/orange hues indicating citation bursts. Links show co-occurrence relationships between keywords across time slices. The early stage (2000–2011) prioritized clinical descriptors. During the growth phase (2012–2017), mechanistic terms emerged. In the modern phase (2018–2025), research converged on neuroimmune interactions and technological integration.

This tripartite analysis elucidates a transformative trajectory from symptom-centric approaches to mechanism-driven precision therapeutics. Emerging frontiers in digital therapeutics and neuroimmune modulation position acupuncture as a critical modality in addressing the projected tripling of global dementia cases.

#### 3.2.7 Literature citation analysis

**Figure 10:**
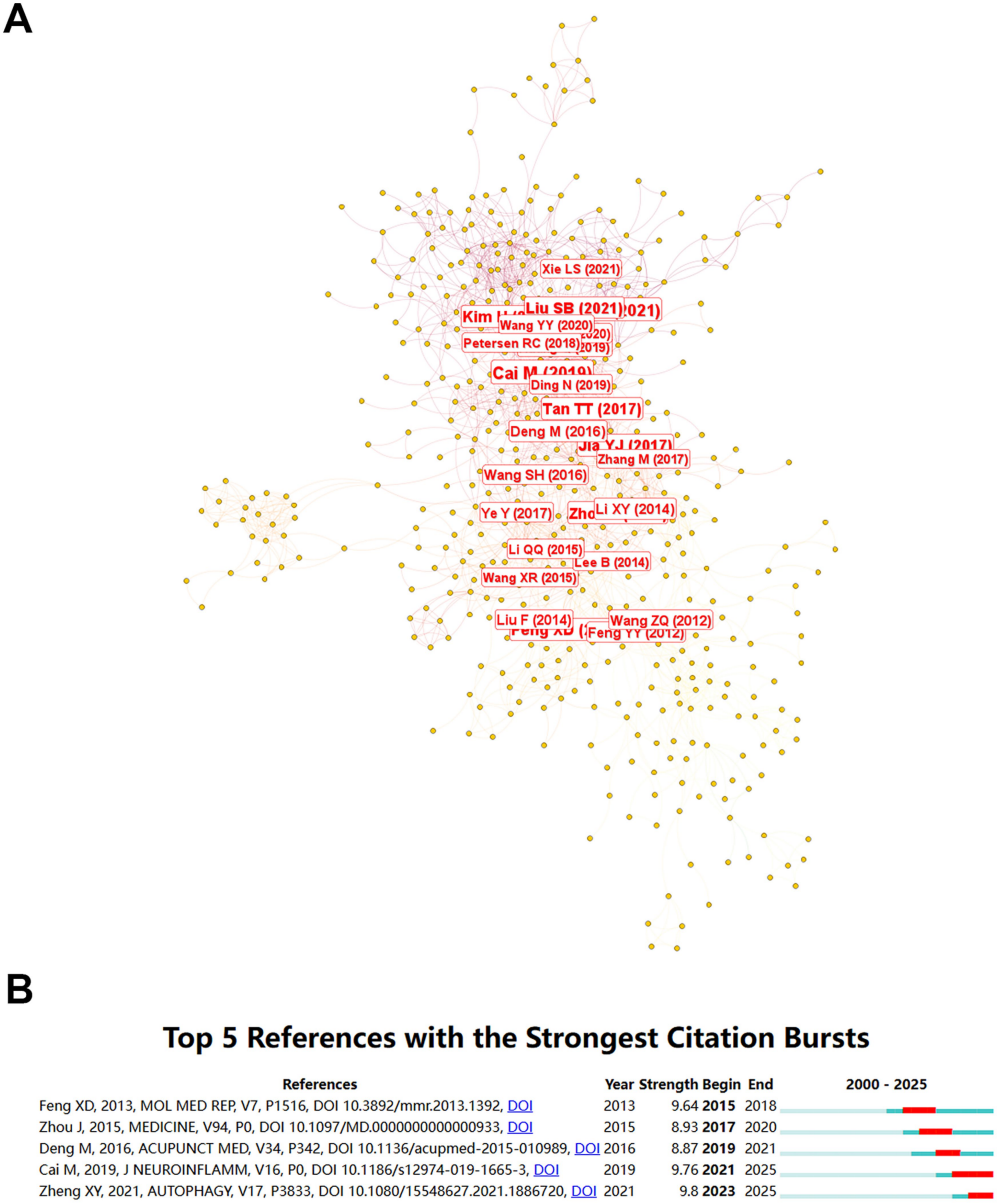
Co-citation network and citation burst analysis of acupuncture research for cognitive impairment. **(A)** A co-citation network map of journals generated using CiteSpace 6.4.R2 based on publications retrieved from the Web of Science database. Nodes represent cited references, with size proportional to their co-citation frequency. Connecting lines indicate co-citation relationships. **(B)** Top 5 references with the strongest citation bursts, identified through burst detection analysis. Citation burst strength (quantified as “Strength”), duration (“Begin–End”), and temporal trends (2000–2025) are annotated.

The co-citation network (Figure 10A), constructed using CiteSpace 6.4.R2 reveals the intellectual foundations and knowledge diffusion patterns in acupuncture-cognitive impairment research. The top five references with the strongest citation bursts were listed in Figure 10B. The first article was published in 2013 by Feng et al. (17), which demonstrated that electroacupuncture at Baihui (DU20) and Shenting (DU24) acupoints ameliorates cognitive impairment in cerebral ischemia–reperfusion injured rats by inhibiting NF-κB-mediated neuronal apoptosis through suppression of pro-apoptotic Bax and Fas expression, thereby reducing infarct volume and improving neurobehavioral outcomes. The second article was published in 2015 by Zhou et al. (12), which demonstrates that acupuncture significantly improves cognitive function (MD = 1.05, 95% CI 0.16–1.93 for MMSE) and activities of daily living in Alzheimer’s disease patients compared to pharmacological interventions, while adjunctive use with donepezil amplifies therapeutic efficacy (MD = 2.37, 95% CI 1.53–3.21), with a favorable safety profile evidenced by only 0.2% incidence of mild adverse events across 3,416 participants. The third article was published in 2016 by Deng and Wang (18), which demonstrates that acupuncture significantly improves cognitive function, as evidenced by higher clinical efficacy rates, Mini-Mental State Examination (MMSE) scores, and picture recognition scores compared to nimodipine alone, and enhances MMSE outcomes when combined with nimodipine in patients with amnestic mild cognitive impairment; however, the conclusions remain tentative due to the low methodological quality of included studies, underscoring the need for larger, rigorously designed trials to validate these findings. The fourth article was published in 2019 by Cai et al. (19), which demonstrates that electroacupuncture (EA) at the KI3 acupoint significantly ameliorates cognitive deficits, enhances synaptic plasticity, reduces neuroinflammation, and attenuates amyloid-*β* (Aβ) deposition in the prefrontal cortex of 5XFAD Alzheimer’s disease mice, primarily by suppressing microglial activation and pro-inflammatory pathways, while restoring glucose metabolism in key brain regions, thereby providing mechanistic insights into EA’s neuroprotective effects against AD-related pathology. The last article was published in 2021 by Zheng et al. (20), which demonstrates that three-needle electroacupuncture (TNEA) significantly alleviates beta-amyloid (Aβ) pathology and cognitive impairment in 5xFAD Alzheimer’s disease mice by activating transcription factor EB (TFEB) via suppression of the AKT-MAPK1-MTORC1 signaling pathway, thereby enhancing autophagy-lysosomal degradation of Aβ and APP/CTFs, reducing neuroinflammation, and restoring synaptic plasticity.

## 4 Discussion

The comprehensive bibliometric analysis presented in this study elucidates the evolving research landscape of acupuncture for cognitive impairment, highlighting distinct thematic trajectories, collaborative dynamics, and translational implications. Our findings underscore the growing academic interest in acupuncture as a therapeutic modality for cognitive disorders, while also revealing critical gaps and opportunities for advancing mechanistic understanding and clinical applications. Below, we contextualize these findings within the broader framework of current research paradigms and propose actionable directions for future investigations.

The divergent thematic foci observed between Chinese and English literatures reflect complementary yet distinct approaches to understanding acupuncture’s therapeutic mechanisms. Chinese studies predominantly emphasize vascular mechanisms, oxidative stress modulation, and pharmacological synergies (e.g., nimodipine), aligning with traditional pathophysiological models of cognitive impairment. This focus is exemplified by high-frequency keywords such as “oxidative stress” and “vascular dementia” suggesting a strong emphasis on cerebrovascular health and redox balance. For instance, electroacupuncture (EA) at GV20 (Baihui) and GV24 (Shenting) has been shown to enhance cerebral blood flow and mitigate oxidative damage in ischemic stroke models, likely through upregulation of antioxidant enzymes (e.g., SOD, CAT) and suppression of lipid peroxidation. These findings align with the mechanistic emphasis on vascular and oxidative pathways in Chinese research. In contrast, English-language literature prioritizes neuroinflammatory pathways, gut-brain axis interactions, and advanced neuroimaging biomarkers (e.g., functional connectivity). The emergence of keywords such as “neuroinflammation,” “gut microbiota,” and “synaptic plasticity” underscores a paradigm shift toward molecular and systemic mechanisms. Recent studies demonstrate that acupuncture attenuates neuroinflammation by suppressing microglial activation (e.g., inhibiting NF-κB and NLRP3 inflammasome pathways) and modulating gut microbial diversity, which in turn regulates peripheral and central immune responses. Furthermore, the activation of transcription factor EB (TFEB) via suppression of AKT-MAPK1-MTORC1 signaling, as evidenced in 5xFAD mouse models, highlights acupuncture’s role in enhancing autophagy-lysosomal degradation of amyloid-*β* (Aβ) and tau aggregates—a novel mechanistic frontier with profound implications for Alzheimer’s disease therapeutics.

These disparities may stem from multifaceted drivers spanning cultural and institutional domains, which warrant systematic exploration to contextualize the observed epistemological bifurcation. In terms of cultural and philosophical foundations, Chinese emphasis on integrative medicine, deeply rooted in traditional Chinese medicine (TCM) principles, likely underpins the focus on vascular and oxidative pathways. TCM conceptualizes cognitive impairment as a manifestation of systemic imbalances, such as “Qi stagnation” or “blood stasis,” prompting investigations into acupuncture’s circulatory-modulating effects (e.g., enhancing cerebral blood flow via GV20 stimulation). This holistic paradigm aligns with national policies promoting TCM integration into modern healthcare systems, fostering research that validates traditional mechanisms through contemporary methodologies. Conversely, Western biomedical frameworks prioritize reductionist approaches, emphasizing molecular and cellular pathways. This dichotomy is exemplified by the surge in English studies exploring neuroinflammation and gut microbiota—domains aligned with modern neuroscience’s emphasis on mechanistic specificity and biomarker-driven validation. With regard to funding priorities and institutional agendas, the “Healthy China 2030” strategy: promote the vigorous development of non-drug therapies in TCM, allowing them to play a unique role in the prevention and treatment of common diseases, frequently occurring diseases, and chronic diseases. For instance, national grants often support basic research of TCM and promoting the innovative development of acupuncture research. In contrast, Western funding bodies, including the NIH and European Horizon programs, increasingly prioritize translational neuroscience and precision medicine, incentivizing studies that dissect acupuncture’s effects on neuroimmune crosstalk or omics-level metabolic shifts. Such funding trajectories inevitably shape research agendas, steering Chinese scholars toward pragmatic clinical outcomes and Western researchers toward mechanistic novelty.

These mechanistic divergences suggest that acupuncture’s therapeutic effects are mediated through multi-target, multi-pathway interactions, necessitating integrative approaches to bridge traditional and modern paradigms. Future mechanistic studies should leverage omics technologies (e.g., transcriptomics, proteomics) and advanced neuroimaging to map acupuncture-induced neural circuit reorganization and systemic metabolic shifts. Additionally, translational models integrating gut microbiota profiling and neuroimmune axis modulation could unravel the bidirectional communication between peripheral and central mechanisms, offering a holistic understanding of acupuncture’s impact on cognitive function.

The bibliometric analysis reveals a robust clinical research output, particularly in Chinese literature, where acupuncture is frequently combined with pharmacological agents (e.g., donepezil, nimodipine) to enhance cognitive outcomes. Meta-analyses consistently report superior efficacy of combined therapies over monotherapies, with significant improvements in Mini-Mental State Examination (MMSE) scores and activities of daily living. However, the methodological limitations of included trials—such as small sample sizes, insufficient blinding, and heterogeneity in acupuncture protocols—highlight the need for standardized treatment regimens and rigorous trial designs. English-language studies, meanwhile, emphasize randomized controlled trials (RCTs) and objective biomarkers (e.g., fMRI-based functional connectivity, CSF Aβ42 levels) to validate efficacy. The growing focus on “computer-based cognitive rehabilitation” and “acupoint specificity” reflects an integration of digital therapeutics and precision medicine approaches. For example, neuroimaging studies have identified key acupoints (e.g., GV20, KI3) that modulate default mode network (DMN) connectivity and hippocampal neurogenesis, providing empirical support for acupoint selection in clinical protocols. Nevertheless, the scarcity of multinational RCTs and long-term follow-up data remains a critical barrier to global acceptance.

The temporal keyword analysis identifies “neuroinflammation,” “gut microbiota,” and “autophagy-lysosomal pathway” as emerging frontiers, signaling a shift toward precision neuromodulation and systems biology. Preclinical studies have already demonstrated acupuncture’s ability to restore gut microbial homeostasis and enhance blood–brain barrier integrity, suggesting its potential as a regulator of the gut-brain axis. Similarly, the activation of TFEB-mediated autophagy presents a promising strategy to clear pathological protein aggregates in neurodegenerative diseases. Technological innovations, such as AI-driven trial designs and wearable devices for real-time monitoring of acupuncture effects, could further revolutionize this field. For instance, machine learning algorithms could optimize acupoint combinations based on individual metabolic profiles, while blockchain technology might enhance data transparency in multinational trials.

Notably, our systematic literature search employing identical search terms yielded substantially different results and article distribution patterns compared with previous studies (16, 21). This discrepancy may be attributed to several methodological distinctions: First, divergence in exclusion criteria appears to constitute a critical factor - our protocol rigorously excluded 415 dissertation records from CNKI database of initial search returns, whereas the comparator study fails to specify dissertation exclusion criteria in their methodology. Second, technical disparities in CiteSpace analytical parameters may significantly impact result interpretation. Specifically, variations in temporal segmentation strategies (annual vs. quinquennial intervals), network pruning algorithms (Pathfinder vs. Pruning Sliced Networks), and threshold selection criteria (g-index coefficients vs. Top N% retention) could collectively alter keyword co-occurrence network topology and thematic clustering patterns.

This study acknowledges several inherent limitations associated with bibliometric methodologies and database selection biases. First, while the inclusion of both CNKI and PubMed aimed to capture a bilingual (Chinese-English) research landscape, the exclusion of other major databases (e.g., EMBASE, Scopus, or Cochrane Library) may introduce selection bias. For instance, clinical trials indexed in EMBASE or systematic reviews in Cochrane are underrepresented, potentially skewing the thematic distribution and citation networks toward studies prioritized by CNKI and PubMed. This limitation could affect the generalizability of findings, particularly for clinical research trends that are more comprehensively covered in specialized biomedical databases. Second, geographic bias persists despite the dual-database approach. CNKI predominantly indexes Chinese-language studies, which may overrepresent regional research priorities whereas PubMed emphasizes English-language publications aligned with Western biomedical paradigms. Consequently, the underrepresentation of non-Chinese/non-English studies in these databases may obscure global collaborative patterns or emerging innovations reported in other regions. Third, language and publication biases cannot be overlooked. Studies published in journals not indexed by CNKI or PubMed, particularly those in non-English or non-Chinese languages, were excluded, potentially omitting valuable insights from underrepresented regions. Additionally, the reliance on bibliometric tools like CiteSpace introduces methodological constraints, such as incomplete keyword normalization or oversimplification of complex co-occurrence networks. Future studies could mitigate these limitations by incorporating multidisciplinary databases (e.g., Scopus for broader coverage) and employing mixed-methods approaches to triangulate bibliometric findings with qualitative insights. Transparency in database selection criteria and explicit acknowledgment of geographic/thematic biases remain critical for contextualizing the scope and applicability of bibliometric conclusions.

In conclusion, this bibliometric synthesis not only maps the current research landscape but also underscores the transformative potential of acupuncture in cognitive impairment management. By bridging mechanistic insights from oxidative stress and neuroinflammation to clinical validation through rigorous RCTs, acupuncture emerges as a versatile and safe therapeutic modality. However, achieving global translational impact requires addressing methodological limitations, fostering international collaboration, and embracing technological advancements. Future research must prioritize mechanistic depth, clinical precision, and cross-disciplinary innovation to unlock the full therapeutic potential of acupuncture in combating the escalating burden of cognitive disorders.

## Data Availability

The original contributions presented in the study are included in the article/Supplementary material further inquiries can be directed to the corresponding author.

## Supplementary material

The Supplementary material for this article can befound online at: https://www.frontiersin.org/articles/10.3389/fneur.2025.1495191/full#supplementary-material
