# Supplementary figures and images for: A bibliometric analysis of acupuncture treatment and cognitive impairment
Source: Front Neurol. 2025 Jun 6;16:1495191. doi: 10.3389/fneur.2025.1495191 (PMC12178889; doi:10.3389/fneur.2025.1495191)

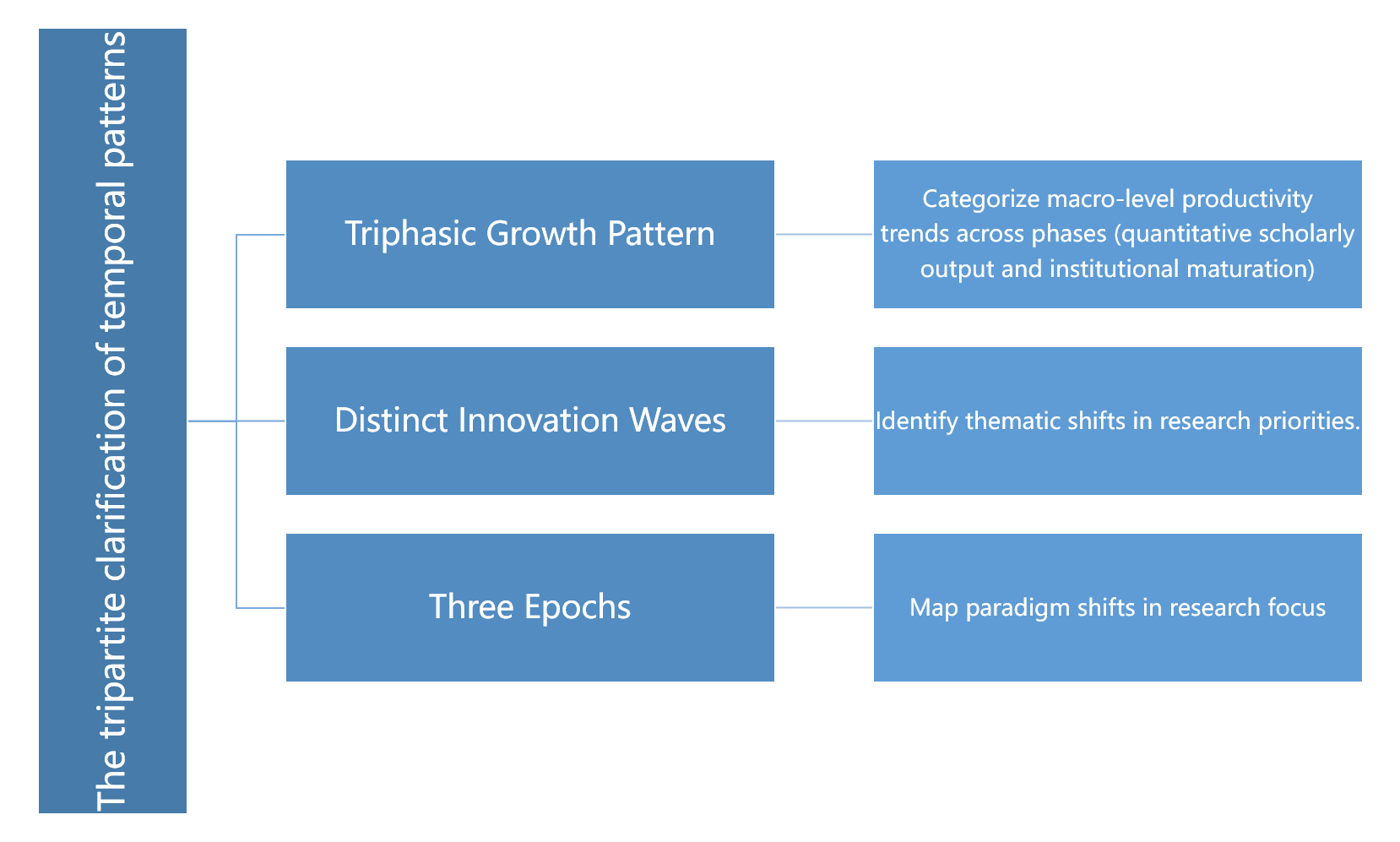

Supplement: Supplementary file 1 [file Supplementary_file_1.TIF]
